# Future Wireless Communication Technology towards 6G IoT: An Application-Based Analysis of IoT in Real-Time Location Monitoring of Employees Inside Underground Mines by Using BLE

**DOI:** 10.3390/s22093438

**Published:** 2022-04-30

**Authors:** Sushant Kumar Pattnaik, Soumya Ranjan Samal, Shuvabrata Bandopadhaya, Kaliprasanna Swain, Subhashree Choudhury, Jitendra Kumar Das, Albena Mihovska, Vladimir Poulkov

**Affiliations:** 1 School of Electronics Engineering, KIIT University, Bhubaneswar 751024, India; sushanta.pattnaik@silicon.ac.in (S.K.P.); jkdasfet@kiit.ac.in (J.K.D.); 2 Faculty of Telecommunications, Technical University of Sofia, 1756 Sofia, Bulgaria; vkp@tu-sofia.bg; 3 Department of Electronics & Communication Engineering, Silicon Institute of Technology, Bhubaneswar 751024, India; 4 School of Physical Sciences, Banasthali Vidyapith University, Rajasthan 304022, India; shuvabrata.bandopadhaya@gmail.com; 5 Department of Electronics & Communication Engineering, Gandhi Institute for Technological Advancements, Bhubaneswar 752054, India; kaleep.swain@gmail.com; 6 Department of Electrical and Electronics Engineering, Siksha ‘O’ Anusandhan, Bhubaneswar 751030, India; subhashree3@gmail.com; 7 Department of Business Development & Technologies, Aarhus University, 8000 Aarhus, Denmark; amihovska@btech.au.dk

**Keywords:** Internet of things (IoT), 5G, beyond 5G (B5G), 6G, industry 4.0, RTLS, artificial intelligence

## Abstract

In recent years, the IoT has emerged as the most promising technology in the key evolution of industry 4.0/industry 5.0, smart home automation (SHA), smart cities, energy savings and many other areas of wireless communication. There is a massively growing number of static and mobile IoT devices with a diversified range of speed and bandwidth, along with a growing demand for high data rates, which makes the network denser and more complicated. In this context, the next-generation communication technology, i.e., sixth generation (6G), is trying to build up the base to meet the imperative need of future network deployment. This article adopts the vision for 6G IoT systems and proposes an IoT-based real-time location monitoring system using Bluetooth Low Energy (BLE) for underground communication applications. An application-based analysis of industrial positioning systems is also presented.

## 1. Introduction

**Figure 1:**
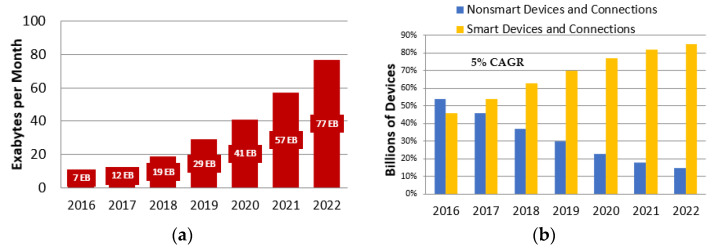
Cisco Annual Report from 2016 to 2022 [2]: (**a**) Cisco Visual Networking Index Global Mobile Data Traffic from 2016 to 2022; (**b**) Global Growth of Smart Mobile Devices and Connections Excluding Low-Power Wide-Area (LPWA).

In recent years, wireless technology has been one of the fastest-growing technologies in the area of communication. Today, wireless technology is becoming one of the largest carriers of digital data around the globe. According to the Cisco Visual Networking Index (VNI) Global Mobile Data Traffic for 2016 to 2022, worldwide mobile data traffic increased about 10-fold over these 6 years, reaching 77 exabytes (approx.) per month by 2022 (Figure 1a [1]). According to [1], the device mix is becoming smarter (advanced computing and multimedia competencies with at least 3G connectivity) with an increasing number of smart devices with high computing capabilities and better network connectivity, which creates a growing demand for smarter and more intelligent networks. The share of smart devices and connections as a percentage of the total will increase from 46 percent in 2016 to 85 percent by 2022, a more than two-fold increase during the figure time frame Figure 1b [1]. It is expected that 75 billion devices will be connected by the end of 2025 [2]. Service providers around the globe are busy rolling out 5G networks to meet the growing demand of the end consumer for greater bandwidth, higher safety and quicker connectivity on the move. Many vendors have additionally begun area trials for 6G and are getting closer to rolling out 5G deployments in the direction of the end of the forecast length.

Moreover, the heterogeneous nature of the next-generation communication networks in terms of the application, communication technology used and involvement of diversified devices brings a large variety of requirements and expectations. Today’s world is focusing more on the IoT due to its wide range of applications from human-centric to industry 4.0/industry 5.0. Nevertheless, device-to-device (D2D), machine-to-machine (M2M) and vehicle-to-vehicle (V2V)/V2X communication technologies constitute the real applications showing the widespread advantages of the IoT [3,4,5,6,7,8,9]. Furthermore, reliable data transmission with low latency is another key challenge for successful IoT applications [10]. The emergence of the Internet of Everything (IoE), which offers remarkable solutions for massive data transmission to the edge network, and the integration of Industrial Control Systems (ICSs) with the IoE recast it as the Industrial Internet of Everything (IIoE) [5]. Again, with the evolution of different emerging technologies such as artificial intelligence (AI), machine learning (ML), cloud computing, cognitive computing, edge computing, fog computing, blockchain technology, etc., various challenges are being addressed in different IoT industrial applications. Such complex IoT networks provide substantial technological prospects that facilitate the realization of good quality of service (QoS) and quality of experience (QoE). For example, the Internet of SpaceThings (IoST) for high speed, reduced latency and umbrella Internet coverage; the Social Internet of Things (SIoT) for an interface between human and social networks; the Internet of NanoThings (IoNT) for telemedicine; and the Internet of UnderwaterThings (IoUT) for improving ocean water quality, cyclonic/tsunami disaster management, etc. [11].

In view of this, the IoE introduces essential protection challenges due to the wide variety of functionality and demanding situations. There is always a dependency of the IoT on cellular networks since long-term evolution (LTE) was introduced, which is enhanced as 5G/6G in some specific scenarios. The demand for high throughput, high energy efficiency and better connectivity with reduced latency time can be attained beyond 5G/6G networks [12]. The 6G system will offer a better enrollment of the IoT devices as the 5G IoT has provided a solid foundation. The future 6G network is envisioned to be service-oriented, where software-defined networks (SDN) and network function virtualization (NFV) will play a vital role in the end-to-end architecture [13]. These technologies are capable of providing better coverage with high throughput, improved spectrum efficiency, greater bandwidth and ultra-low latency. The 6G IoT system is sustainable for high-accuracy localization and sensing, which are necessary for most of the envisioned highly computationally intensive applications.

### Related Work and Key Contributions

A growing number of research works focus on current advances in wireless and IoT technology, including in-depth analysis of the advanced technology concepts, methodology and techniques.

Specifically, [14] provides a comprehensive survey on key enabling technology for 6G, where the emphasis is on a discussion of the operation of the individual technology with useful statistics for industries and academic researchers on the potential for investigating new research directions. The authors of [15] discussed the requirements of 6G and recent research trends to enable 6G capabilities and design dimensions by employing disruptive technologies such as artificial intelligence (AI) and driving the emergence of new use cases and applications manifested by stringent performance requirements. A review of 6G in terms of use cases, technical requirements, usage and key performance indicators (KPI) is presented in [16]. Here, the authors presented a preliminary definition roadmap, specifications, standardization and regulation for 6G. A survey on wireless evolution toward 6G networks is presented in [17], discussing the capabilities of network slicing technology with AI to enable a multitude of services with different quality of service (QoS) requirements for 6G networks. A comprehensive survey on the existing trends, applications, network structure and technologies of 6G is presented in [18], with a focus on industrial markets and use cases of 6G that take advantage of a better on-device processing and sensing, high data rates, ultra-low latencies and advanced AI. In [19], the authors presented an overview of 6G describing the complete evolution path from 1G networks to date and focusing on several key technologies such as terahertz communications, optical wireless communications (OWC) and quantum communications for improving the data rates.

A comprehensive survey on the convergence of the IoT and 6G is presented in [20,21] with a focus on edge intelligence, reconfigurable intelligent surfaces, space–air–ground–underwater communications, terahertz communications, massive ultra-reliable and low-latency communications and blockchain as the technologies that empower future IoT networks. A comprehensive study on 6G-enabled massive IoT is presented in [22], where ML and blockchain technologies are discussed as the primary security and privacy enablers. In [23], the potential of the IoT and 6G for various use cases in healthcare, smart grid, transport and Industry 4.0 have been elaborated jointly with the challenges during their practical implementations. Several shortcomings of 5G and features of 6G related to social, economic, technological and operational aspects such as the weakness of short packet and sensing-based URLLC, which may limit the dependability of low-latency services with high data rates or the lack of support of advanced IoT technologies are discussed in [24]. Current research activities, therefore, should focus on innovative techniques such as advanced time-stamp stream filtering combined with intelligent network slicing to support multi-party (source) data stream synchronization in very low latency environments coupled with distributed control (at the edge).

In [25], the author mainly focuses on the integration of blockchain technology into 6G, the IoTand IIoT networks. Blockchain technology has a strong potential to fulfill the requirements for massive 6G-based IoT for the integrity of personal data protection, data privacy and security and scalability. Furthermore, a sustainable ecosystem-focused business model, driven by blockchain-empowered 6G networks is thoroughly analyzed to deal with the cutting-edge worldwide economic disaster. Envisioning the green 6G–IoT network, a novel joint design technique using intelligent reflective surface (IRS) and ambient backscatter communication (ABC) is proposed in [26]. This method is primarily based on the joint design of an iterative beamforming vector, an IRS phase shift and reflection coefficients to decrease the AP’s transmit power without affecting the QoS. The author in [27] addressed the three fundamental components, i.e., artificial intelligence (AI), mobile ultra-high speed and the (IoT) for the future 6G network. The authors focused on the recent approaches, research issues and key challenges of IoT network topology and terahertz (Tz) frequency. A comprehensive survey of existing 6G and IoT-related works is summarized in Table 1.

The contributions to this paper can be outlined as follows:

- We present the vision of the IoT with the technologies impacting it with their key features
- We review several applications and challenges of the IoT in different domains.
- We present different connectivity standards of the IoT and a rigorous review of these technological standards
- We present a comparative analysis between 5G and 6G.
- We present the vision and key features of 6G with its different aspects.
- We present a brief review of several challenges of 6G.
- We propose a BLE-based real-time location monitoring system by using the IoT

**Figure 2:**
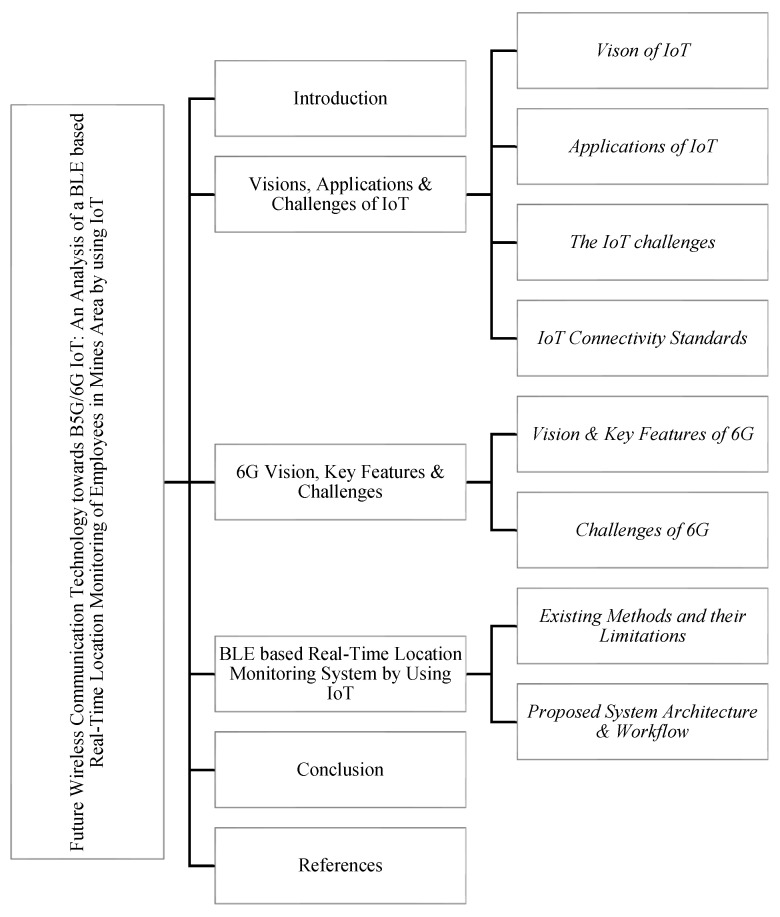
Structure of the Paper.

The remainder of this article is organized as follows. Section 2 presents the vision, applications and challenges of the IoT, including the connectivity standards and a comparative analysis of their capabilities. In Section 3, a comparative analysis of 5G and 6G with the vision key features and the challenges of 6G is presented. Section 4 proposes and discusses a BLE-based real-time location monitoring system by using the IoT. Finally, we draw conclusions in Section 5. Related abbreviations are listed in the Appendix. A schematic representation of the structure of the paper is shown in Figure 2.

## 2. Visions, Applications and Challenges of the IoT

In the last few decades, the IoT has become the most promising and thriving area of research in academia and industry. The IoT extends the existence of communication by converging clients, businesses and industries by connecting intelligent things with each other through the cloud. These smart connections encompass different network applications, communication technologies and smart devices along with physical and virtual things. The IoT paradigm is a transformation from a centralized computer-based network to a completely distributed network of smart devices. To take the potential benefits of the IoT and to compete globally, the IoT European Research Cluster (IERC) has focused mainly on establishing a cooperation platform between companies and organizations for developing more research activities on the IoT at the European level. The primary objective of IERC is to facilitate making the research activities more ambitious and neoteric. The International Telecom Union (ITU) was the first international agency to produce a report on the IoT in 2005 [28]. Thereafter a new standard of the IoT was approved by the ITU in 2012 [29]. However, the term IoT was first used by the Massachusetts Institute of Technology’s (MIT’s) Kevin Ashton in 1999 [30].

### 2.1. Vison of the IoT

**Figure 3:**
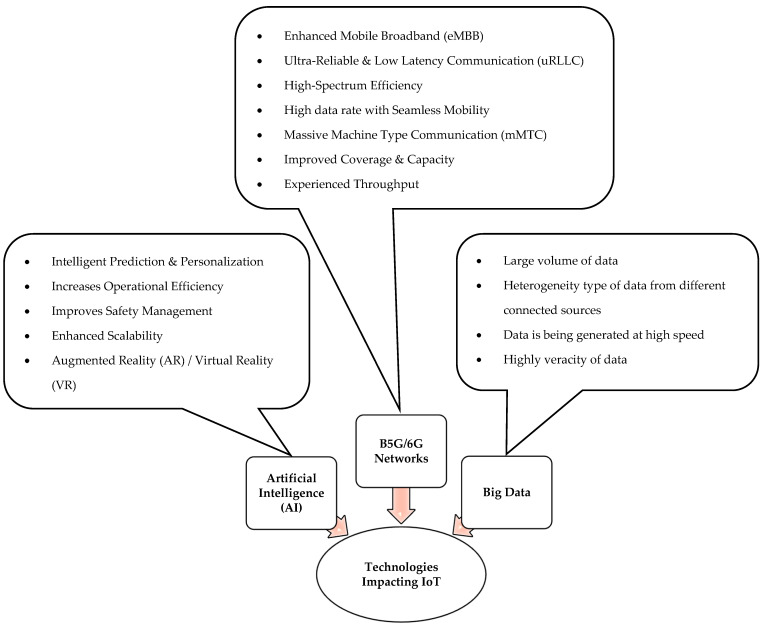
Technologies impacting the IoT.

The vision of the IoT has different perspectives based on the data generated by the connected objects and the technology used. During the early stages of IoT implementation, the vision was to identify the physical objects by using radio frequency identification (RFID) tags. However, due to recent technological advances, the vision of the IoT has been reformed by encapsulating varying technologies and smart sensors. The IoT leads the way in unfolding the new generations of different compelling applications and services in the field of Industrial IoT (IIoT), Industry 4.0 and Society 5.0. Figure 3, illustrates the key technologies that impact the IoT. 

### 2.2. Applications of the IoT

IoT applications in various sectors have been assessed based on their impacts on society and the economy along with their technology readiness level (TRL). The applications of the IoT are diversified based on their use in different fields such as intelligent homes, healthcare, agriculture, transportation, the environment, education, retail and logistics, industries and many more [31,32,33,34]. Consequently, the IoT has also had an impact during the pandemic era of COVID-19 in many aspects, e.g., contact tracing, virus detection by temperature scanning, remote health monitoring, quarantine e-tracking, virus spread control, etc., and also in tackling the post-COVID-19 situation [35,36,37,38]. AI-integrated IoT technology for the early detection of COVID-19 is discussed in [37]. This research mainly focuses on analyzing the extracted features of cough, shortness of breath and speech difficulties by using long short-term memory (LSTM) with recurrent neural network (RNN). In [38], an IoT-based real-time learning system is developed to control the spread of COVID-19 infection in the context of smart healthcare for residents. The system is used to monitor and analyze user activities and environmental parameters which helps predict critical cases, so alerts can be sent to the caretakers. A few applications of the IoT are briefly presented in Table 2.

### 2.3. The IoT Challenges

With an increase in the number of smart devices and real-time applications, the complexity of IoT networks has increased in terms of their densities and architecture. These complexities scale down the performance competencies of the current IoT network. There are several IoT challenges, namely, universal standardization, connectivity, cloud computing, energy efficiency, IoT protocol and architecture in addition to security and privacy. The IoT is still in its developing stage; so many more challenges have to be addressed with the revolution of technologies in the future research domains of the IoT. A few challenges of the IoT are briefly presented in Table 3.

### 2.4. IoT Connectivity Standards

**Figure 4:**
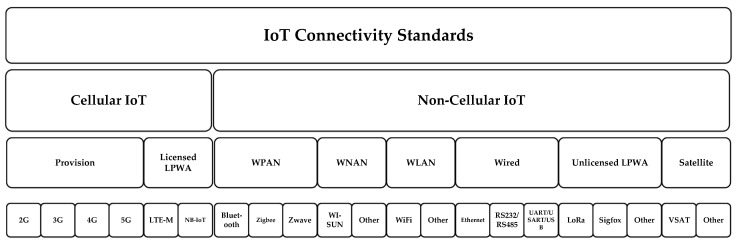
IoT Connectivity Standards.

As per the IoT analytics report, there are mainly 21 IoT connectivity standards that can be broadly classified in two ways: as cellular IoT and non-cellular IoT connectivity standards. The cellular IoT standards are operated at a licensed spectrum, whereas the non-cellular IoT is operated at a non-licensed spectrum. Different IoT connectivity standards are depicted in Figure 4 [135] [Source: IoT Analytics Report 2021]. A comparative analysis of different IoT connectivity standards is presented in Table 4.

## 3. Vision, Key Features and Challenges of 6G

With the standardization of 5G about to complete and its commenced global deployment, several latent limitations to meet the necessary requirements of IoT systems still remain. These impediments mainly relate to the high computation, security, wireless brain-computer interface (WBCI) intelligent communication in terms of more autonomous human-to-machine (H2M) communication, holographic communication (augmented reality/virtual reality) and AI. These data-hungry applications require more spectrum bandwidth (e.g., mm-wave) and high spectral efficiency which can be realized at the sub-terahertz (sub-THz) and THz bands [154]. Furthermore, due to the incorporation of a wide variety of mobile applications, there are some more challenges (beyond uRLLC, coverage, localization, privacy, power consumption, better quality-of-service, etc.) that need to be addressed in the future B5G wireless communication standards. In this context, the 6G is attracting more researchers from academia and industries towards itself. A comparative analysis between 5G and 6G is presented in Table 5.

### 3.1. Vision and Key Features of 6G

**Figure 5:**
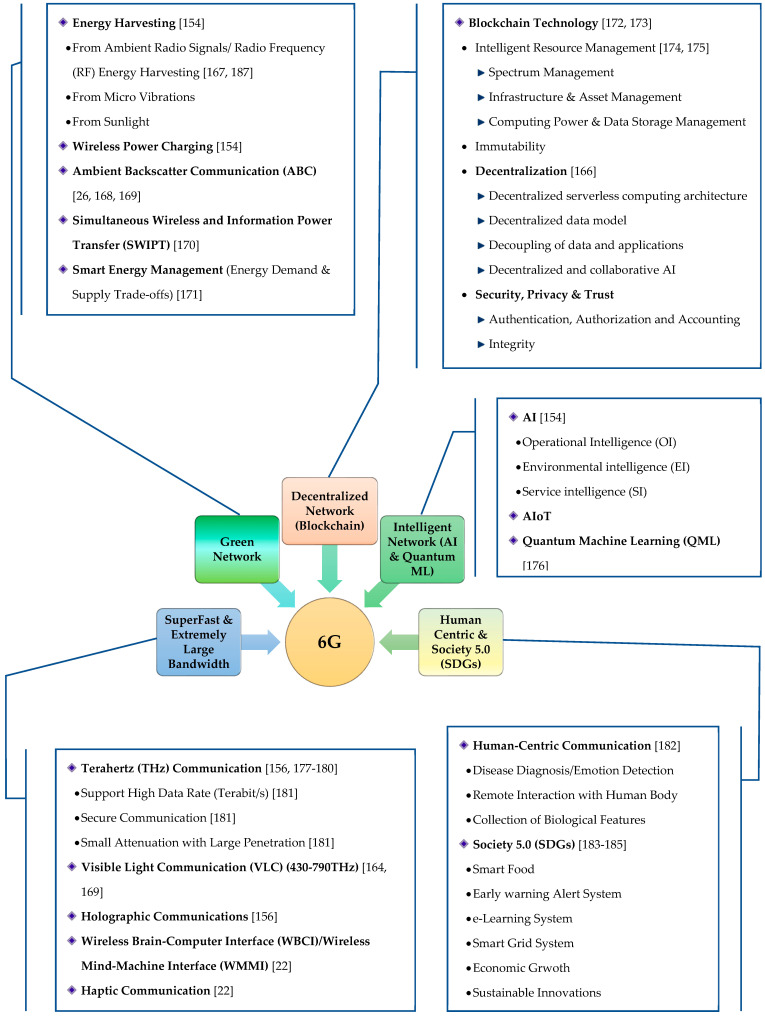
Vision and key features of 6G [22,26,154,156,165,166,167,168,169,170,171,172,173,174,175,176,177,178,179,180,181,182,183,184,185,186].

Despite the dramatic revolution of IoT–5G application in today’s wireless networks, 6G is anticipated to excel 5G in many ways, not only in daily life, but also in Society 5.0. Even though 6G is not a talking point of global harmony so far, some additional features with more potential and capabilities are being discussed. In this section, a comprehensive vision of a 6G network is presented from multiple perspectives as shown in Figure 5.

#### 3.1.1. Intelligent Network

As 6G is envisioned as a fully automated and smart network, the incorporation of AI, MLand quantum machine learning (QML) makes the future wireless networks more intelligent and predictive by limiting human efforts [176,187]. AI and ML are the transforming technologies and data analytics tools in the modern era of wireless communication that bring new research challenges in the field of 6G IoT [186]. By using big data and ML, a more precise performance prediction model can be implemented in a 6G IoT network to make smart decisions for security, optimization, resource allocation, network management, self-organization, etc., [155,156,157,158,159,160,161,162,163,164,165,188]. Due to the high veracity/volume data and complex 6G IoT network structure, it is necessary to instigate more futuristic learning/training frameworks for high-dimension neural networks (HDNN) [165].

#### 3.1.2. Decentralized Network

Due to the emergence of multi-access edge computing (MEC) in the 5G network, there are several limitations in the centralized network, e.g., privacy, security, trust, incompatibility of the existing protocol to the dynamic connectivity and distributed and ubiquitous computing [166]. Thus, it is necessary to prepare a blueprint of decentralized architecture to support such a dynamic and autonomous network. In this regard, blockchain is a promising technology for the future 6G network and is capable of dealing with these challenges. Blockchain technology can provide a decentralized network management framework that can be used for resource management, data sharing/storage, spectrum sharing and other challenges [172,173,174,175,189].

#### 3.1.3. Green Network

The 6G network is expected to meet the essential requirements for energy-efficient wireless communication globally. The green 6G network enables minimum energy utilization and helps achieve a peak data rate (THz) during signal transmission. A significant improvement in the energy efficiency of a network can be greatly experienced by incorporating different energy-harvesting techniques [154,190]. This also helps facilitate green communication by reducing CO_2_ emission. In addition, several communication techniques, e.g., D2D communication, massive multiuser multiple-input-multiple-output (MIMO), heterogeneous network (HetNet), green IoT, non-orthogonal multiple access, energy-harvesting communications, etc., may be adopted to facilitate green communication for future wireless networks [191,192,193].

#### 3.1.4. Superfast Network

With reference to the data analysis shown in Figure 1, the ever-increasing demand for high data rate and seamless connectivity to such ultra-dense networks can be provided by integrating terahertz (THz) (ranges from 0.1–10 THz) communication in 6G networks [168,177,178]. A vast amount of unused radio spectra which can be efficiently used to increase network capacity is available in the THz band. THz is additionally reasonable for high data rate transmission and short-range communication by empowering the ultra-high bandwidth and uLLC paradigms. An extensive review of THz communication with its future scope and challenges is presented in [194].

#### 3.1.5. Human-Centric

It is believed that human-centric communication is a key feature of the 6G network. With the help of this technology, sharing and/or accessing different physical features can be possible by humans. To accelerate human-centric communication rather than technology/machine-centric communication, the principal means of human perception must be incorporated into the communication system module [195]. A human-centric communication framework needs two fundamental aspects—technology and user experience (UE). The latter includes human behavior as well as psychological and socioeconomic contexts and needs to be considered during the modeling and analysis of the communication system [183,184,195].

In 2016, Society 5.0 was initiated by the Japanese cabinet in its Fifth Science and Technology with a vision to build a “Super Smart Society” [196]. Later, the vision was revised and presented by the Keidanren Business Federation with the prime focus of delivering sustainable development goals (SDGs) through the creation of Society 5.0 [183,184,197]. Society 5.0 is designed to solve different social issues by taking advantage of technological advancements. Considering different aspects of economic growth, social and environmental conditions, 17 primary objectives and 167 goals are listed in the Agenda 2030 by the United Nations to address several global challenges [198,199].

### 3.2. Challenges of 6G

**Figure 6:**
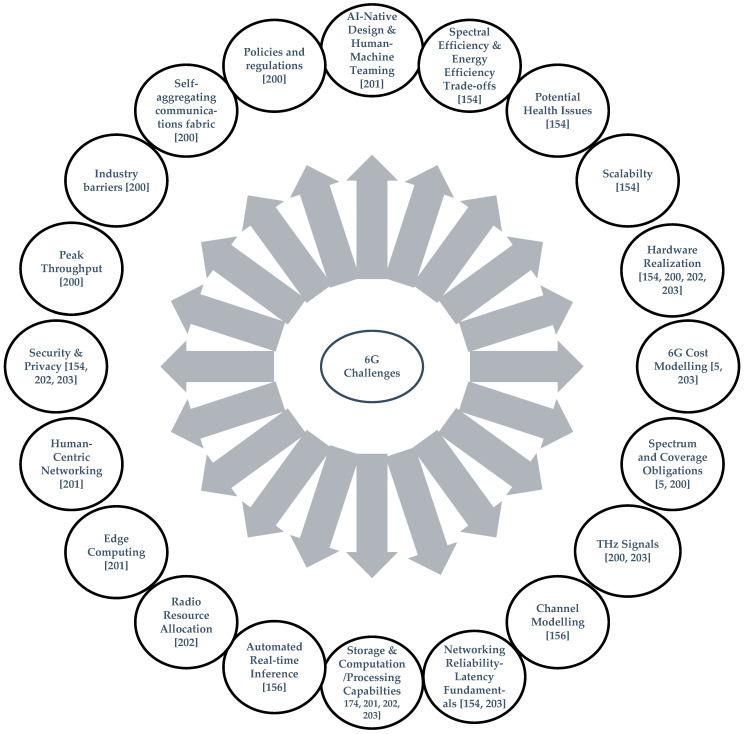
Challenges of 6G [5,154,156,200,201,202,203].

Even though several advanced features have been added to 6G networks to enhance the performance matrices in comparison with 5G networks, there are still some key challenges that must be addressed further. These challenges are broadly classified into two categories: (i) technological challenges that include high throughput, EE, connectivity flexibility, more intelligent optimization techniques, etc., and (ii) non-technological challenges including industry barriers, spectrum allocation, regulatory policies and standardization, etc. [200]. A few key challenges of the future 6G networks are summarized in Figure 6.

In addition, due to the integration of the IoE, terrestrial and non-terrestrial communication networks in 6G, their different heterogeneous highlights must be considered to productively coordinate them. Heterogeneity is likewise present in the protocol that those communication networks will comply with. Thus, 6G is taking on the massive task of integrating a number of heterogeneous aspects [203]. Furthermore, due to the inclusion of mm-Wave and THz communication, 6G networks are facing several more open challenges, e.g., more sensitive low-power transmitter, new model architecture, advanced propagation techniques for better coverage and directional communication. The networks must also deal with system noise, channel fading and fluctuations [169,203,204,205]. Several more challenges such as computational and processing resources due to the application of AI [206], a few ML application-related challenges [207], training issues and interoperability challenges [208], challenges in estimating the channel information by using reconfigurable intelligent surfaces (RIS) [209,210] and computational and trade-off challenges due to the application of artificial neural networks (ANN) in the IoT [211] have been recognized for the future 6G networks.

## 4. An IoT-Based Real-Time Location Monitoring System by Using BLE

Mining is one of the most speculative businesses around the globe. Most of the mines all over the world are lagging in different safety measures causing many casualties and deaths. The basic causes of death in underground mines are gas accidents, rock falling, ventilator accidents, fire, explosions, etc. Considering the safety issues of the employees/workers inside the mines, real-time location tracing of those employees becomes a major concern. Effective underground communication is necessary to collect more information about the mines or workers. However, there are various constraints while collecting the real-time data inside the mines such as restricted transmitting power, large attenuation of the transmitted signal from the rock wall and low penetration of the electromagnetic signal. In this regard, it is always beneficial to take the potential advantages of low-power and short-range communication technologies such as, RFID, Zigbee, Bluetooth, Bluetooth low energy (BLE), etc.

In this section, a scenario for a Bluetooth low energy (BLE) beacon-based real-time location monitoring of employees/workers by using the IoT is presented. A BLE beacon and microcontroller are used to design this asset-tracking product and have been implemented in the IoT here by connecting this device to the cloud.

### 4.1. State-of-Art

Underground communication inside mines is a major factor for the safety and security concerns of the mineworkers. The advent of IoT technology and its usefulness can be beneficial for the mining industry. It is believed that a robust communication infrastructure using IoT technology inside the mines may enhance the safety of the workers and is also capable of providing real-time information resulting in quick action to avoid lethal situations. Several researchers have proposed various frameworks and ideas for efficient communication inside the mines based on IoT technology, which includes low-power and short-range communication.

The authors of [212,213] proposed a wireless sensor network (WSN)-based monitoring system for underground mines. In this proposed technique, various sensors are placed at different locations to collect activities and positions of the employees, and the collected data are transferred to the end user or the central server via BS. Nageswari et al. [214], proposed an IoT-based smart mine monitoring system that uses radio frequency (RF) technology for communication purposes inside the mines. With this proposed technique the real-time location and real-time sensing of the dynamically varying environment can be achieved by using RF technology and WSN network, respectively. The major drawback of this proposed model is that large-signal transmission loss occurs through the walls of underground mines. An IoT-based mine safety system using WSN was proposed in [215,216]. In these proposed techniques, the authors used a Zigbee module for information collection from the cloud and measured the surrounding parameters of underground mines with the help of various sensors. A mine safety system using WSN was proposed in [217], where the authors constructed a prototype by using Zigbee and WSN to monitor safety issues and to measure the ambient properties, e.g., temperature, humidity, airflow, etc., inside the underground mines. A Zigbee compliant RFID-based safety system for underground coal mines was proposed in [218], where a unified wireless mesh-network infrastructure was used to monitor and locate the workers and measure the different environmental parameters inside the coal mines. Similarly, an IoT-based system for underground coal mines that uses a microcontroller, a node MCU and various sensors to measure the environmental conditions and safety measures of workers was proposed in [219]. A LoRaWAN-based coal safety and health monitoring system was proposed in [220]. In this proposed methodology, LoRaWAN uses low-power RF with a wide communication range and IoT technology for monitoring the workers’ health and observing the status of the circumstances in the coal mines.

There are several existing technologies used for communication purposes in underground mines. The most common approaches are RFID, Zigbee, Bluetooth, GPS, etc. Table 6 presents a comparative analysis of some existing technologies in terms of their pros and cons [214,215,216,217,218,219,220,221].

### 4.2. Proposed System Architecture and Workflow

**Figure 7:**
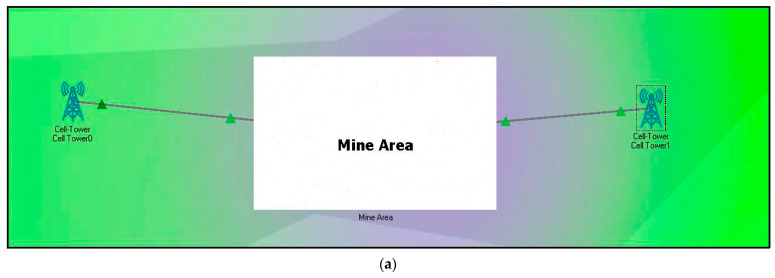

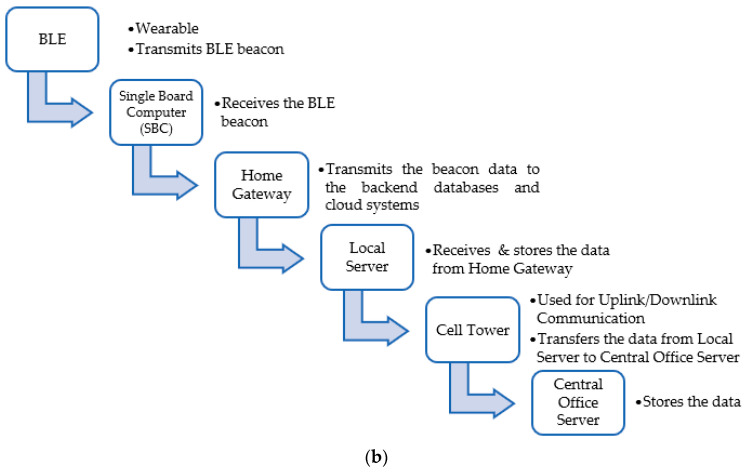
Proposed system architecture and workflow. (**a**) Proposed System Model; (**b**) Complete Workflow Process.

To overcome these issues, our proposed technique uses BLE, which is a low-power and low-cost technology. This proposed methodology reduces the deployment cost and complexities by using the BSs of the existing cellular network infrastructure for the communication process. The system architecture of BLE-based real-time location monitoring in mines by using the IoT is shown in Figure 7. In this scenario, two base stations (BS) are deployed to provide necessary services (uplink/downlink) to the BLE devices through the central office server as shown in Figure 7a, and the complete workflow is shown in Figure 7b.

**Figure 8:**
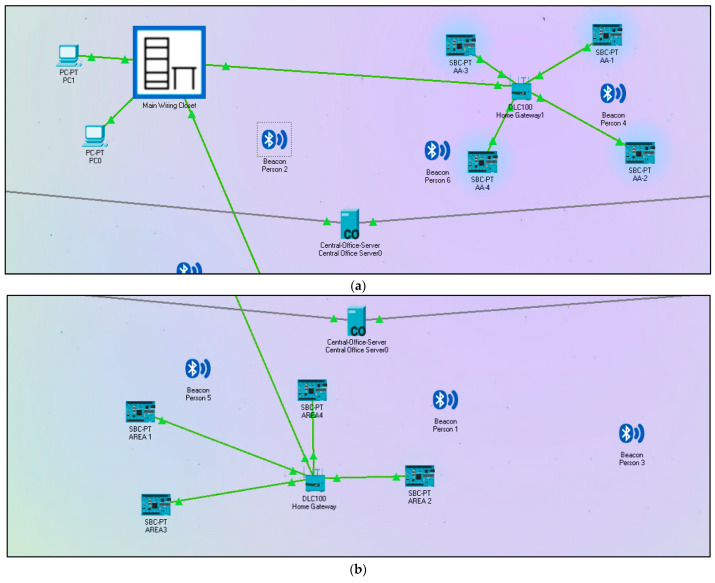
Position of SBC and random distribution of BLE devices inside the mine area (**a**,**b**).

Figure 7a shows the coverage area of BLE and cell towers based on their transmitted power. The blue and green colored portion shows the energy region of BLE devices and cell towers, respectively. As can be seen, cell tower 0 transmits more power compared to cell tower 1. All the BLE devices are wearable or are attached to the employee working inside the mines. In this proposed method, beacons are considered because they can be easily identified by single board computers (SBC) as shown in Figure 8. Different beacons are accessed by the nearest SBC based on their coverage area. The blue-colored region indicates the transmitted energy by the beacon signal as shown in Figure 8a. The system contains beacons that are small and inexpensive, which emit signals in the same fashion as BLE. The used beacons have a short-range and can triangulate position in the same way that a phone uses cell towers with an assisted global positioning system (AGPS). These transmitters are deployed at known points inside the mines, and they permit the device to obtain area fixes. This data can be utilized to make new client encounters, for example, turn-by-divert headings for indoor situating from gateways/applications that read the guide signals.

The scenario presented in Figure 8a,b shows the position of the SBC (fixed position) and a random distribution of BLE devices, as the position of BLE device (wearable) depends on the position of the employee working inside the mine. The BLE receivers/gateway receives the universally unique identifier (UUID) transmitted by the beacons in a repetitive manner as shown in Figure 8a,b. These signals can be utilized to differentiate between subgroups and individual ones in the subgroups. It is modified to check the accessible BLE signals “on the air” and the received signals contain the accompanying snippets of data in a bundle size of 60 bits, with 10 bits specifying major and minor values. The received signal strength indicator (RSSI) values can be utilized to decide the distance of the receiver to every one of the reference points. As those region statistics are stored inside the database, navigation of the receiver also can be tracked, and alerts can be generated if certain rules are violated. All the beacon data are stored in a local server through the gateway and then transferred to the central office server through cell towers as shown in Figure 8a,b. The central office server is continuously updated based on the real-time information sent by the BLE beacon through the gateway. This information can be used to find the real-time location of the employees/workers inside the mines.

### 4.3. Simulation Result and Discussion

**Figure 9:**
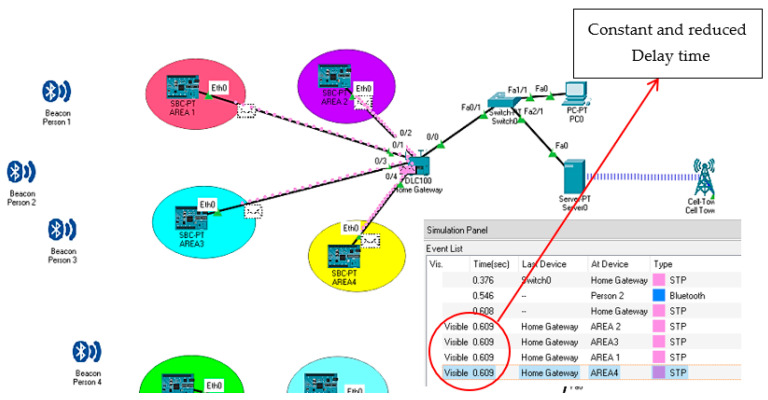
Discovery time of BLE device.

The simulation result in Figure 9 shows the discovery time of the BLE devices. It can be seen that the visibility time of the BLE device is constant, and the delay time is also very small. Hence, it helps to find the real-time location of the employees/workers inside the mines within a short time. Due to the small visibility time, the rescue process can be improved for the employees/workers (real-time locations) inside the underground mines during any hazardous situation.

## 5. Conclusions

This paper summarizes and relates the future direction of IoT applications to current 6G trends, development sand challenges. The study looked at the vision and different technologies impacting the IoT as outcomes of international research. The paper considered the applications in various sectors and provided a summary of the different IoT technologies. The various IoT connectivity standards and a few challenges remaining open for IoT integration with cellular systems were outlined. The IoT is a basic building block for next-generation industrial standard 4.0/5.0 smart applications in home, city, agriculture, healthcare and many more uses, but this requires a major upgrade of the physical and network layers of upcoming cellular wireless networks. In this paper, a brief comparison between 5G and 6G was presented in terms of the technical features. The vision and key features of 6G along with the implementation challenges were discussed. This paper also includes a case study related to the real-time application of the IoT to locate the employees in underground mines using BLE technology. The system architecture and workflow for the given application were presented. This article might assist the researcher apprehend various challenges with their applications of the IoT and 6G to the real world.

## Figures and Tables

**Table 1:** A Comprehensive Survey of existing 6G and IoT related works.

References	Authors	Year	Research Topic	Objectives/Key Contributions
[14]	Alsabah et al.	2021	Concept on 6G Network	A comprehensive review fn 6G-enabling technologies with a short discussion on their principle of operations, applications, current researchand challenges.
[15]	Shahraki et al.	2021	Enabling technologies and future challenges for 6G	A brief discussion on the enabling technologies, requirementsand trends of 6G with a focus on challenges and recent research activities, including tactile Internet and terahertz communication.
[16]	Jiang et al.	2021	Roadmap definition and Key Performance Indicators of 6G	A comprehensive survey on 6G use cases, architecture, key drivers, enabling technologies, etc.
[17]	Nasir, et al.	2021	Evolution of intelligent 6G network	A review on the evolution of wireless technology toward 6G, focusing on the key driving forces behind the shift. A short discussion on network slicing technology with AI to facilitate multimode services with varying QoS.
[18]	Hakeem et al.	2022	6G applications and future research	A brief discussion on trends, regulations, industrial marketsand analysis of 6G requirements in terms of network architecture and hardware–software design.
[19]	Qadir et al.		6G-IoT concept	A brief survey on 6G networks, research activities, key enabling technologiesand case studies with the main focus given to the discussion of terahertz communication and visible light communication.
[20]	Nguyen et al.	2022	6G-enabled IoT networks	A holistic review of the convergence of 6G and IoT networks with a brief discussion on the key enabling technologies for the IoT including terahertz communication, reconfigurable intelligent surfaces and blockchain. A few research challenges and applications of the IoT are also discussed in depth.
[21]	J. H. Kim	2021	Recent trends in 6G related to IoT technology	A short discussion on key drivers, enabling technologiesand current research trends of 6G with a brief introduction about viable applications of 6G to the IoT.
[22]	Guo et al.	2021	6G-enabled massive IoT	A survey on the key drivers and requirements for IoT-enabled applications with several constraints of 5G are also highlighted. A case study on fully autonomous driving is presented to manifest the support of 6G to massive IoT. A few key technologies such as ML and blockchain technologies are also discussed.
[23]	Barakat et al.	2021	Opportunities of 6G in IoT technology perspective	A comprehensive review of the IoT use cases based on its wide variety of implementations.
[24]	Mahdi et al.	2021	Road map from 5G to 6G	A holistic review of 5G and 6G technologies in terms of energy, he IoTand ML.
[25]	Jahid et al.	2021	Integration of blockchain technology with 6G and Ithe IoT	A comprehensive survey on integrity, privacyand security issues, with the mitigation techniques encountered in blockchain-integrated 6G cellular networks.
[26]	Liu et al.	2021	6G green IoT network	A novel method of minimizing the access point’s transmitting power is introduced by implementing the ABC and IRS technique jointly.
[27]	Ndiaye et al.	2022	IoT network topology and 6G communication technology	A brief discussion on the fundamental components of a 6G network. A short overview of key challenges and research issues of IoT network topology and terahertz frequency

**Table 2:** Applications of the IoT.

Focused Area	Applications	References
Intelligent Home	Facilitating comfortable lifestyle Helps in reducing the carbon footprint of energy consumption Intrusion detection QoS-based services Design of sensitive home automation system Indoor monitoring	[39,40,41,42,43,44,45,46]
Smart Cities	Analyze and predict the performance of applications used in scalable platforms Location finding along with the updated location configuration features Smart energy Smart mobility and traffic management Digital forensics Smart governance Smart healthcare Smart education	[41,43,47,48,49,50,51,52,53]
Medical and Health Care	Health and fitness monitoring Remote medical diagnostics Wearable electronics gadgets Patient monitoring Disease management system to improve reliability Mobile medical home monitoring system to improve the rapidity of factor measurements Human factor evaluation in information exchange in the healthcare environment Integration of AI in clinical medicine	[35,36,50,51,54,55,56,57,58,59,60]
Environment	Ecological habitat monitoring Weather monitoring CO_2_ Emission monitoring Collection of recyclable materials Smart disaster management system The revival of a rural hydrological/water monitoring system Smart environment Water environment monitoring	[50,51,61,62,63,64,65,66,67]
Agriculture	Automated irrigation control Green house control Precision agriculture field operation and evaluation Smart farming Aquaponics farming Smart precision farming Livestock farming Smart decision-making system for real-time analysis Integration of AI in monitoring and management	[62,64,67,68,69,70,71,72,73,74]
Transport	Optimal route finding Smart traffic Vehicular speed monitoring Toll fee collection Information about busy traffic Smart parking Surveillance monitoring Automated/Driverless vehicle ML-enabled smart transport	[48,49,75,76,77,78,79]
Retail and Logistics	Smart payments through near field communication (NFC) and Bluetooth Stock management Shipment monitoring Cargo handling and tracking Remote vehicle diagnostics Supply chain management	[77,78,80,81,82,83,84,85]
Industry	Machine diagnosis and prognosis Indoor air quality monitoring Manufacturing automation Industrial blockchain technology IIoT for low-power wide-area networks (LPWANs) Smart factories	[33,86,87,88,89,90,91,92]

**Table 3:** Challenges of the IoT.

Focused Area	Challenges	References
Constrained Resources	Limited manufacturing techniques for small size and low-cost device resources Spectrum resources scarcity for IoT enabling technologies Smart antenna	[93,94,95,96,97,98]
Scalability, Reliability and Interoperability	Self-addressing, discovering and classification Host identification and address mapping Interoperability and availability Lack of efficient and reliable communication by using TCP (transmission control protocol)/UDP (user datagram protocol) protocol Unreliable packet delivery Lack of interoperability between different protocols	[96,99,100,101,102,103,104,105]
Privacy and Security	Integrity, validation, authentication and trust Data and physical device security Confidentiality Cyclic redundancy check (CRC) Message authentication code (MAC) Limitations of symmetric cryptography and public–key cryptography operation Different IoT threats such as fragmentation attack Poor encryption	[35,36,96,101,104,106,107,108,109,110,111,112,113,114,115,116,117,118,119]
Big Data and Cloud Computing	Lack of computational resources Low data storage Loss of data packets Optimization of multi-objective functions Edge computing Liability sensitization toward redundant tasks Centralized data acquisition system Must support domain-specific programming	[104,105,107,108,120,121]
Universal Standardization	For technology and other regulatory For communication among heterogeneous devices Protocol standardization Spectrum harmonization	[95,96,120,122]
Connectivity	Supportiveness of tactile Internet and multimedia communications High data rate applications, e.g., AR and VR Reduced latency for real-time applications Fast and précised localization determination Good QoS Signaling overhead on edge devices Seamless connectivity Internetworking Wide range of connectivity Gossip-based algorithm for better connectivity for poor communication network	[95,104,117,120,123,124,125]
Energy Efficiency	Energy harvesting Energy efficient (EE) LPWANs Self-sustainability of machines due to limited energy Power losses and energy conversions EE MAC and cross-layer protocols Technologies for green IoT Intelligent energy management Energy saving solutions for network softwarization	[95,96,107,126,127,128,129,130,131,132]
IoT Architecture and Protocol	Autonomous and incremental computation framework/architecture Flexible and open architecture for heterogeneous devices More intelligent self-organizing network (SON) Efficient management of radio resources, service provisions, orchestration, etc. Integration with AI Traditional business model Mobility management Simple, light efficient security protocol Efficient risk management Efficient radio access protocol Efficient tracking and protection management in cloud environment	[95,96,104,105,107,122,133,134]

**Table 4:** Comparison of different IoT Connectivity Standards.

Standards	Range of Communication	Max. Data Rate	Frequency Spectrum Used	Power Consumption	Standardization	Modulation	Multiplexing/ MAC Scheme	Security Algorithm
NFC	0.1 m [136]	106–848 Kbps [136]	13.56 MHz [34,136]	Low (<40 mA) [136]	ISO/IEC 14443, 18092 JIS X6319-4 [136]	ASK, BPSK [136]	TDMA [137]	Encryption Cryptographic, Secure Channel, Key Agreements [136]
Bluetooth	0–10 m [138]	24 Mbps [138]	2.4 Ghz [138]	10 mw [12], 2.5–100 mW [139]	IEEE 802.15.1 [140]	GFSK, DQPSK, 8DPSK [138,140]	TDD [138], FHSS [140]	E0, E1, E21, E22, E3, 56–128 bit [140]
BLE	50 m [89], 70 m [136]	1 Mbps [136,140]	2.4 Ghz [140]	Low (<12.5 mA) [140]	IEEE 802.15.1 [140]	GFSK, FHSS Star [136]	FHSS [140]	AES-128 [140]
ANT	<30 m [140]	1 Mbps [140]	2.4 Ghz [140]	Low (<16 mA) [140]	Proprietary [140]	GFSK [140]	TDMA [140]	AES-128, 64 bit [140]
Zigbee	10–300 m [138]	20–250 Kbps [138]	ISM Bands 2.4 GHz/915 MHz (USA)/868 MHz (EU) [138]	Medium (1 mw-100 mw) [141]	IEEE 802.15.4 [140]	BPSK (868–915 MHz) O-QPSK (2.4 GHz) [138,140]	DSSS [89], CSMA/CA TDMA + CSMA/CA [138]	AES-128 [138,140]
Zwave	100 m [136], 0–30 m [138]	9–100 Kbps [136], 40 kbps [138]	2.4 GHz 908.4 MHz (USA) 868.4 MHz (EU) [138]	Medium (1 mW) [141]	Proprietary [140], ITU G.9959 [142]	FSK, GFSK [136,137,140]	FHSS [89], CSMA/CA [138]	AES-128 [138,140]
WiFi	10–100 m [138]	65 Mbps [138]	ISM Bands 2.4–5 Ghz [138]	Low to Medium (32–200 mW) [138,139]	IEEE 802.11 [143]	BPSK, QPSK, COFDM, CCK, M-QAM [138]	CSMA/CA + PCF [138]	CCMP 128 [138]
LoRaWAN	5–20 km [144]	50 kbps [144]	Unlicensed ISM bands (868 MHz in Europe, 915 MHz in North America and 433 MHz in Asia) [144]	Low (10.5–28 mA) [145]	LoRa Alliance [143]	LoRa CSS [143,146,147,148]	Pure—ALOHA [146,147,149]	AES-128 encryption [146,147]
NB-IoT	1–10 km [144]	204.7–234.8 Kbps [136], 200 kbps [144]	Licensed LTE frequency Bands [136,144]	Low (46 mA) [150]	3GPP [136,143]	QPSK [143], BPSK [147], GFSK, BPSK [136]	OFDMA for downlink and SC-FDMA for uplink [151]	3GPP 128–256 bit [136,144,146]
Sigfox	10–40 km [136,144]	100–600 bps [136], 100 bps [144]	Unlicensed ISM bands (868 MHz in Europe, 915 MHz in North America and 433 MHz in Asia) [136,144]	Low (10–50 mA) [145]	Sigfox [143]	BPSK [92], DBPSK for Uplink and Gaussian frequency shift keying (GFSK) for downlink [136,147,148]	R-FDMA [152,153]	AES-128 encryption [147,148]

**Table 5:** A comparative analysis between 5G and 6G.

Parameters	Technological Standards	
	5G	6G
*Frequency Band*	Sub 6 GHz, 30–300 GHz [155]	Sub 6 GHz, 30–300 GHz, 0.3–3 THz [155]
*Average Data Rate*	100 Mbps [155]	1 Gbps [155]
*Latency*	1 ms [155]	<1 ms [155]
*Mobility*	≥500 kmph [155,156]	≥1000 kmph [155,156]
*Maximum Channel Bandwidth*	1 GHz [156]	100 GHz [156]
*Connection Density*	$10^{6}\;\mathrm{devices}/{\mathrm{km}}^{2}$ [156]	$10^{7}\;\mathrm{devices}/{\mathrm{km}}^{2}\;$[156]
*Reliability (Packet Error Rate)*	$10^{-5}$ [156]	$10^{-9}$ [156]
*Area Traffic Capacity*	$10\;\mathrm{Mbps}/m^{2}$ [155,156]	$10\;\mathrm{Gbps}/m^{2}$ [155,156]
*Service Types*	eMBB, mMTC, uRLLC [155]	mbRLLC, muRLLC, HCS, MPS [155]
*Multiplexing*	CDMA [157,158], OFDM, GFDM [158], FBMC [159], Adaptive Time–Frequency Multiplexing [160]	Smart OFDMA + Index Modulation, OMA [161], NOMA [161], OAM [162], Spatial Multiplexing [163]
*Power Consumption*	Low to Medium	Ultra-low [164]
*Downlink Spectral Efficiency*	30 bps/Hz [165]	100 bps/Hz [165]
*Energy Efficiency Gains in Comparison With 4G*	10× [165]	1000× [165]
*Network Architecture*	Centralized [155]	Decentralized [155,166]

**Table 6:** A comparative analysis of some existing technologies.

Technology	Advantages	Disadvantages
GPS	*Large coverage area*	*Inefficient for underground mines*
GSM	*Large coverage area*	*Communication delay exists*
RFID	*Non line-of-sight* *Communication, High Penetration, Compact Size*	*High maintenance of RFID tags, Low Security*
RF TECHNOLOGY	*Non line-of-sight* *Communication*	*High penetration loss/ Signal attenuation is very high*
RADAR	*Accurate and High Penetration*	*High CapEx and OpEx*
ZIGBEE	*Low Power Consumption, Low Latency Time, Cheap*	*Low Penetration, Poor non-interference*
BLUETOOTH	*Low Power Consumption, Low Latency Time*	*High CapEx and OpEx, Small coverage area*

## Data Availability

No new data were created or analyzed in this study. Data sharing is not applicable to this article.

## Abbreviations

3GPP	3rd Generation Partnership Project
ABC	Ambient Backscatter Communication
AGPS	Assisted Global Positioning System
AI	Artificial Intelligence
ANN	Artificial Neural Networks
AR	Augmented Reality
BS	Base Stations
BLE	Bluetooth Low Energy
CapEX	Capital Expenditure
CDMA	Code-Division Multiple Access
CRC	Cyclic Redundancy Check
D2D	Device-to-Device
EE	Energy efficient
eMBB	Enhanced Mobile Broadband
FBMC	Filter-bank Multicarrier
GFDM	Generalized Frequency-Division Multiplexing
GPS	Global Positioning System
GSM	Global System for Mobile Communication
H2M	Human-to-Machine
HCS	Human-Centric Service
HDNN	High Dimension Neural Networks
HetNet	Heterogeneous Network
ICS	Industrial Control System
IERC	IoT European Research Cluster
IIoT	Industrial Internet of Things
IoE	Internet of Everything
IoNT	Internet of NanoThings
IoST	Internet of SpaceThings
IoT	Internet of Things
IoUT	Internet of UnderwaterThings
IRS	Reflective Surface
ITU	International Telecom Union
KPI	Key Performance Indicator
LoRaWAN	Long Range Wide Area Network
LPWA	Low-Power Wide-Area
LPWAN	Low-Power Wide-Area Networks
LSTM	Long Short-Term Memory
LTE	Long Term Evolution
M2M	Machine-to-Machine
MAC	Message Authentication Code
mbRLLC	Mobile broadband RLLC
MEC	Mobile Edge Computing
MIMO	Multiple-Input-Multiple-Output
MIT	Massachute Institute of Technology
ML	Machine Learning
mMTC	Massive Machine Type Communication
MPS	Multipurpose 3CLS and energy services
MTC	Machine-type Communicaiton
muRLLC	Massive uRLLC
NFC	Near Field Communication
NOMA	Non-Orthogonal Multiple Access
OAM	Orbital Angular Momentum
OMA	Orthogonal Multiple Access
OFDM	Orthogonal Frequency-Division Multiplexing
OpEX	Operational Expenditure
OWC	Optical Wireless Communications
QML	Quantum Machine Learning
QoE	Quality of Experience
QoS	Quality of Service
RADAR	Radio Detection And Ranging
SBC	Single Board Computer
RF	Radio Frequency
RFID	Radio Frequency Identification
RIS	Reconfigurable Intelligent Surfaces
RNN	Recurrent Neural Network
RSSI	received signal strength indicator
RTLS	Real-Time Location monitoring System
SDGs	Sustainable Development Goals
SIoT	Social Internet of Things
SON	Self-Organizing Network
SWIPT	Simultaneous Wireless and Information Power Transfer
TCP	Transmission Control Protocol
THz	Terahertz
TRL	Technology Readiness Level
UDP	User Datagram Protocol
uRLLC	Ultra-Reliable Low Latency Communication
UUID	Universally Unique Identifier
V2V	Vehicle-to-Vehicle
VR	Virtual Reality
VNI	Visual Networking Index
VLC	Visible Light Communication
WBCI	Wireless Brain-Computer Interface
WLAN	Wireless Local Area Network
WMMI	Wireless Mind-Machine Interface
WNAN	Wireless Neighborhood Area Network
WPAN	Wireless Personal Area Network
WSN	Wireless Sensor Network
